# Mapping the Neuropsychiatric Symptoms in Alzheimer’s Disease Using Biomarkers, Cognitive Abilities, and Personality Traits: A Systematic Review

**DOI:** 10.3390/diagnostics15091082

**Published:** 2025-04-24

**Authors:** Athanasios Chatzikostopoulos, Despina Moraitou, Vasileios Papaliagkas, Magda Tsolaki

**Affiliations:** 1 Laboratory of Psychology, Department of Cognition, Brain and Behavior, School of Psychology, Faculty of Philosophy, Aristotle University of Thessaloniki (AUTh), 54124 Thessaloniki, Greece; demorait@psy.auth.gr; 2 Laboratory of Neurodegenerative Diseases, Center of Interdisciplinary Research and Innovation (CIRI-AUTH), Balcan Center, Buildings A & B, 57001 Thessaloniki, Greece; tsolakim1@gmail.com; 3 Greek Association of Alzheimer’s Disease and Related Disorders, 54643 Thessaloniki, Greece; 4 Department of Biomedical Sciences, School of Health Sciences, International Hellenic University, Alexandrion University Campus, 57400 Thessaloniki, Greece; vpapaliagkas@gmail.com; 5 1st Department of Neurology, School of Medicine, Faculty of Health Sciences, Aristotle University of Thessaloniki, 54636 Thessaloniki, Greece

**Keywords:** Alzheimer’s disease, neuropsychiatric symptoms, biomarkers, cognition, personality

## Abstract

**Background/Objectives:** Symptoms (NPS) in Alzheimer’s disease (AD) have multiple effects in daily living, not only for the patients but for their caregivers too. The present systematic review was performed in order to identify if biomarkers, cognitive functions, and personality traits can be considered as important factors for the development and maintenance of these symptoms. **Methods:** To achieve that, the existing literature spanning the period from 2018 to 2024 was critically analyzed. To be included in the review, a study had to investigate any of the factors mentioned above. In total, 182 articles were assessed for eligibility, and 50 met the inclusion criteria. **Results:** Most of the studies were focused on the role of biomarkers and found that amyloid β, tau and phospho-tau protein are closely related to the incidence and the severity of NPS. In fewer studies, cognitive function and personality traits were also associated with NPS. **Conclusions:** In conclusion, biomarkers, cognitive function and personality traits are associated with NPS, but the underlying mechanisms, still, mostly remain unknown.

## 1. Introduction

Alzheimer’s disease (AD) is the most common neurodegenerative disease, characterized by memory loss, deficits in other cognitive functions, and behavioral changes that can ultimately affect daily functioning. The main neuropathological hallmarks of the disease are the accumulation of amyloid plaques and neurofibrillary tangles, which lead to synaptic loss and, ultimately, neurodegeneration [1].

AD diagnosis has been based mainly on clinical manifestations, making it a possible or probable diagnosis rather than a confirmed one. However, in recent years, the development of biomarkers has succeeded in measuring in vivo AD pathophysiology. These biomarkers can be found in blood, in cerebrospinal fluid (CSF) or with the use of Positron Emission Tomography (PET). The identified biomarkers are the amyloid β (Aβ) (A) deposition, the tau protein (T), and the neurodegeneration (N), creating an unbiased descriptive classification scheme [2]. The severity of brain changes is linked to the level of cognitive decline a person experiences [3].

However, AD is not a normal part of aging. According to the World Health Organization, healthy aging is not merely the absence of disease, but rather a lifelong process of maximizing functional abilities to support well-being in older age [4]. Nevertheless, older adults are usually experiencing the preclinical AD “stage” of Mild Cognitive Impairment (MCI), which is characterized by objectively measured deficits in one or more cognitive domains, as demonstrated by standardized neuropsychological testing, while functional independence in daily life is preserved [5]. MCI presents in various subtypes, including amnestic MCI, primarily affecting memory, and non-amnestic MCI, affecting other cognitive functions. These subtypes are further categorized as either single-domain or multi-domain, based on whether a single cognitive function or multiple cognitive functions are impaired, respectively [6]. A meta-analysis of 41 cohort studies found annual conversion rates of MCI to AD dementia of 8.1% and 6.8% in specialist and community settings, respectively [7]. Besides these findings, Davis et al. (2018) found that annual transition probabilities from MCI to more severe states at age 65 were 8% for normal cognition, 22% for MCI due to AD, and 25%, 36%, and 16% for mild, moderate, and severe AD dementia, respectively [8]. The likelihood of progression increased with age, whereas a positive correlation was observed between age, AD severity, and the rates of institutionalization and mortality.

Neuropsychiatric symptoms (NPS) are common in dementia and can be challenging to treat due to their wide range and complex causes. The most common categories of NPS include agitation, aggression, irritability, mood disorders, anxiety, hallucinations, delusions, sleeping disturbances, and eating disorders [9]. The development of NPS is likely caused by a complex interplay of factors. Central to this process is neurodegeneration, which damages circuits involved in emotions, actions, motivation, or perception. This damage can directly trigger NPS or make individuals more susceptible to environmental factors. Additionally, NPS may occur due to challenges individuals face in adjusting to their surroundings as their cognitive abilities decline. Other potential causes include unmet needs, acute illnesses that cause confusion, or environments and caregiving that are not suited to the patient’s current abilities [10]. NPS can have serious consequences if not managed effectively. They can accelerate disease progression, interfere with daily activities, reduce quality of life, and increase caregiver burden and healthcare costs. In severe cases, NPS may lead to hospitalization or admission to a long-term care facility [11].

It is known that the frequency and the severity of NPS increase with the deterioration of cognition, and, for this reason, they may constitute early manifestations of AD [12]. Specifically, 30% of AD cases show NPS in advance of a remarkable cognitive decline [13]. Neuroimaging biomarkers show that grey and white matter structural atrophy is associated with a higher frequency of NPS, especially psychosis, agitation, and apathy [14]. As the disease progresses to MCI, NPS such as agitation, sleep disturbances, and irritability become more frequent and have been linked to an increased risk of progression to dementia [15]. Neurobiological studies indicate that NPS in MCI correlate with amyloid-beta and tau accumulation, neuroinflammation, and structural brain changes affecting the fronto-limbic circuits. The presence of NPS across these early disease stages suggests that they could serve as biomarkers for early diagnosis and targets for intervention to slow the progression to AD [16].

We still do not fully grasp the exact connection between neuropsychiatric symptoms and cognitive impairment. Proposed mechanisms include four possible pathways, which are not mutually exclusive but could potentially work together in various ways: (1) an etiologic pathway, where NPS might initiate pathophysiologic brain changes that ultimately lead to AD pathology, (2) a common neuropathological pathway, where NPS and cognitive deficits might originate from damage to the same brain regions, (3) a psychological reaction, as individuals become aware of their declining cognitive abilities and may experience emotional distress like depression or anxiety, and (4) a combination of neuropsychiatric symptoms and biological factors that might accelerate cognitive decline [17]. Moreover, Young et al. (2018) reported a consistent association between premorbid neuroticism and the development of NPS. In contrast, premorbid conscientiousness, extraversion, openness, and agreeableness may be predictive factors for the development of NPS in the future [18]. These findings suggest that the potential predictive value of various personality traits requires further investigation using more standardized and comprehensive methodologies.

Furthermore, several non-biological factors can contribute to the development of NPS [19]. A large percentage of people with dementia experience NPS triggered by undiagnosed medical issues such as urinary tract infections, thyroid problems, anemia, constipation, and pneumonia [20]. Moreover, drug interactions and inadequate assessment or treatment of pain can lead to NPS [21], or they can be an expression of unmet needs (physical, psychological, social, or emotional) [19]. A lack of communication and activity, either due to verbal difficulties, lack of environmental stimuli, or the negative styles of a carer’s communication, can also lead to NPS [22,23]. However, the present study does not focus on these non-biological factors, because we considered, as the first crucial step, to clarify—at least to some extent—the potential biologically defined substrate that can be associated with specific NPS in AD, something that seems complicated enough in the extant literature.

### Objectives

Specifically, the primary objective of this systematic review is to provide a comprehensive analysis of the role of AD-related biomarkers, cognition, and personality traits in the development and maintenance of each of the main NPS in AD. The study is guided by research questions such as: “What is the role and potential interplay of specific biomarkers, cognitive functions, and personality traits in the development of each of the main NPS in AD? Are there any differences in the biologically defined substrate which can be associated with the specific NPS? Are there any common biologically defined factors that can be associated with all or many of the NPS in AD?”. The present, PRISMA-compliant, systematic review aims to critically analyze recent studies published between 2018 and 2024, summarizing how these factors contribute to specific NPS development in AD. Through a thorough examination of existing literature, we seek to clarify whether specific biomarkers, cognitive functions, and personality traits can help explain challenging behaviors in AD.

## 2. Materials and Methods

### 2.1. Search Strategy

The systematic review was conducted in accordance with PRISMA (Preferred Reporting Items for Systematic Reviews and Meta-Analyses) guidelines for the reporting of systematic reviews and meta-analyses [9]. Literature research was conducted in December of 2024 in PubMed (MEDLINE), Scopus, Semantic Scholar, and Google Scholar using the following keywords, which had to be part of the title, abstract, or keywords: (“neuropsychiatric symptoms” OR “behavioral symptoms” OR “psychological symptoms”) AND (“Alzheimer’s Disease” OR AD) AND biomarkers AND (cognition OR “cognitive functions” OR “cognitive abilities”) AND (personality OR “personality traits”).

### 2.2. Eligibility Criteria

Inclusion criteria for the review required studies to investigate at least the relationship of NPS with AD biomarkers using blood, CSF, or PET. Only recent studies published in English between 1 January 2018 and 20 July 2024 were considered, because there were somewhat similar systematic reviews before. Studies published in English were selected to facilitate accurate comprehension and analysis. The timeframe was also selected to capture the most current research, particularly regarding the increasing integration of biomarkers in diagnostic procedures of AD. The present review focused on primary research studies to ensure the inclusion of original data and analysis. Consequently, previous reviews, meta-analyses, editorials, and chapters were excluded.

### 2.3. Study Selection

The online database search yielded 135 potential studies. Duplicate removal reduced this to 98 records, which were subsequently screened. Of these, 22 were excluded due to being some type of review, and 30 were excluded for lack of relevance to the aim of the study and the research questions. The excluded studies looked at biomarkers related to AD pathology but did not measure or correlate them with NPS. Consequently, 49 studies met the eligibility criteria and were included in the final analysis. The Newcastle–Ottawa Scale was used to assess the quality of included studies. Scores were assigned for selection, comparability, and outcome/exposure. Only studies with scores above 5 were included in the final analysis (see Appendix A).

### 2.4. Data Synthesis

A qualitative synthesis was performed to integrate the findings of the included studies. To improve the presentation of the results, the data were organized according to the type of the design of the study (longitudinal or cross-sectional), in order to detect whether there were any systematic differences in the findings of the two types of studies regarding the role of specific biomarkers, cognitive functions, and personality traits, as well as their interplay, in the development of NPS (see Table 1).

At a second step—after the presentation of the results related to potential common factors and their interplay that can be associated with all or many of the NPS in AD, as these factors are revealed from longitudinal and cross-sectional studies separately—the data were organized on the basis of each NPS, in order to reveal potential specific biologically-defined substrates that can be associated with specific NPS. At this point, it should be mentioned that the present review does not aim to reveal causal relationships between the factors of interest and NPS. Rather, it aims to represent one of the first trials to detect patterns of associations between specific biologically defined factors and specific NPS in AD. This scientific trend, very recently, seems to have already begun to give some interesting research data.

## 3. Results

### 3.1. The Included Papers

**Figure 1:**
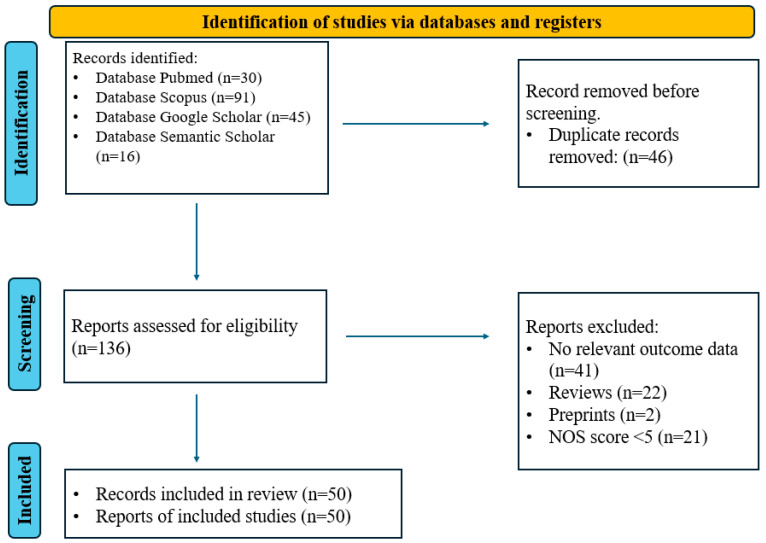
PRISMA 2020 flow chart for the selection of articles included in the systematic review.

This review included 50 studies [24,25,26,27,28,29,30,31,32,33,34,35,36,37,38,39,40,41,42,43,44,45,46,47,48,49,50,51,52,53,54,55,56,57,58,59,60,61,62,63,64,65,66,67,68,69,70,71,72,73] that investigated the role of AD-related biomarkers, cognitive abilities, and personality traits in the development of NPS. The literature search and selection process, adhering to PRISMA 2020 guidelines [74], are depicted in Figure 1. Table 1 provides a detailed summary of the included studies, including sample sizes, study designs, objectives, measures, biomarkers, and key findings.

### 3.2. Characteristics of the Included Studies

Findings from 50 studies included in the review suggest that several biologically defined variables may be responsible for the development and occurrence of NPS (Table 2). However, the relative contribution of each variable appears to vary according to study design, disease stage, and methodological quality. While cross-sectional studies tend to confirm concurrent associations, longitudinal studies yield stronger evidence regarding predictive and dynamic associations. All retrieved articles were written in English, and notably, there was considerable heterogeneity in the sample sizes and the diagnosis of the participants. The majority of studies included patients with dementia and MCI, whereas others included SCI patients and healthy participants. This variability reflects the natural trajectory of Alzheimer’s disease and related dementias. The use of different populations is not a significant bias, but it aligns with the increasingly accepted view of dementia as a continuum, where biological changes precede clinical symptoms by years. Moreover, longitudinal studies involving early-stage participants (cognitively unimpaired or MCI) facilitate the identification of NPS as prodromal features of dementia, rather than consequences of cognitive decline [26,41].

The NPS in most of the studies were measured with the NPI scale, which is the most widely used neuropsychological tool for the measurement of NPS over the past 25 years. It consists of 12 domains, each containing questions, sub-questions, and ratings of frequency and severity. These domains assess hallucinations, delusions, anxiety, depression, apathy, irritability, agitation, disinhibition, elation, aberrant motor disturbances, appetite/eating behaviors, and night-time sleep disturbances. Moreover, it has been translated into approximately 40 languages, and it is recognized as a valid and reliable tool [75].

To assess cognitive functions, a variety of neuropsychological tools were used. Most studies in this review utilized the Mini-Mental State Examination (MMSE) and Montreal Cognitive Assessment (MoCA) to measure global cognition and the Clinical Dementia Rating (CDR) scale to evaluate functional abilities. To measure executive functions, Trail Making Tests A and B were mainly used. Additionally, specialized neuropsychological tests were used to examine specific cognitive functions, depending on each study’s objectives and expected outcomes.

For the assessment of personality, the NEO Five-Factor Inventory (FFI) was mainly used. It is a standardized psychometric tool developed to assess the five broad personality factors according to the Five-Factor Model: neuroticism, extraversion, openness to experience, agreeableness, and conscientiousness. It contains 60 items, with 12 items per trait. As it is widely validated, its brevity, ease of administration, and strong psychometric properties, such as high internal consistency and construct validity, make it useful in large-scale or longitudinal studies, including those focused on aging and neurodegenerative diseases. In dementia research, the NEO-FFI has been useful for the assessment of premorbid personality traits that potentially modulate vulnerability to NPS, cognitive decline, or disease progression. However, as a self-report measure, it may be biased, especially when it is administered to individuals with cognitive impairment [76].

For the evaluation of biomarkers, CSF samples were mainly used and specifically amyloid β (Aβ), tau, and p-tau protein. Some studies also examined these biomarkers in plasma. More recent studies used PET (amyloid or FDG) to have more precise measurements of biomarkers and their role in specific brain regions. Additionally, MRI was frequently used to assess structural changes in the brain because of neurodegeneration.

### 3.3. Findings Related to NPS in General and to Specific Ones

#### 3.3.1. Findings Related to NPS in General

Studies examining NPS globally, often measured with the total NPI, identified biomarkers related to amyloid and tau pathology as key contributors. Lower CSF Aβ42 levels and reduced Aβ42/Aβ40 ratio were repeatedly associated with higher total NPI scores, suggesting a relationship between amyloid burden and the severity of NPS [26,38,44,45,48,49,59,60,70,73]. Longitudinal studies further demonstrated that reduced Aβ42, in combination with elevated total tau and p-tau, predicted the worsening of total NPI scores over time [26,41]. Furthermore, tau/Aβ42 and p-tau/Aβ42 ratios were associated with general NPS burden and progression [26,56,59].

Several studies highlighted the role of neuroinflammation and neurodegeneration biomarkers in NPS. Increased levels of inflammatory markers such as interleukin-6 (IL-6), C-reactive protein (CRP), and soluble intercellular adhesion molecule-1 (sICAM-1) were observed in individuals with higher total NPI scores, indicating systemic inflammatory involvement [33,39]. Moreover, glial activation, measured either through plasma GFAP or PET-based microglial imaging, was associated with higher NPI scores, demonstrating the relevance of innate immune activation in the behavioral phenotype of AD [62,64]. Structural neuroimaging studies supported these findings, linking gray matter atrophy in the hippocampus, anterior cingulate cortex, and medial frontal regions with more severe NPS profiles. In addition, several studies identified widespread white matter damage, particularly in the cingulum, fornix, and frontal associative tracts, as correlates that could be associated with higher NPI scores, suggesting that disrupted connectivity contributes to behavioral dysregulation [59].

Functional imaging provided mixed results. While some PET studies reported associations between increased amyloid or tau binding and general NPI severity [30,40], others found weaker or non-significant links—particularly for amyloid—when tau burden, inflammation, or connectivity disruption were also considered [38,51]. Notably, Dang et al. [55] concluded that tau PET outperformed both amyloid and FDG-PET in predicting total NPI scores, pinpointing the significance of tau pathology in the neurobiology of NPS. Across studies, longitudinal imaging data offered stronger evidence for predictive relationships between biomarker changes and behavioral trajectories compared to cross-sectional associations.

Cognitive impairment also played a role in NPS expression. While findings varied across studies, global cognitive decline and executive dysfunction were the most commonly recognized domains [26,41,53,54,68]. Deficits in cognitive flexibility, attention, and memory were associated with higher total NPI scores, suggesting that broad cognitive dysfunction may amplify or reflect underlying behavioral disturbances. Importantly, longitudinal analyses showed that baseline cognitive impairments could precede increases in NPI scores, supporting a model in which cognitive symptoms and NPS evolve concurrently but are also bidirectionally reinforcing over time [27,33,41].

Only five studies explored the role of personality traits in modulating overall NPS burden. The findings suggest that higher neuroticism may increase general vulnerability to behavioral disturbance, while conscientiousness, extraversion, and openness may have protective effects [30,43]. A longitudinal study by Ronat et al. [43] showed that individuals with higher emotional instability displayed not only higher total NPI scores but also greater hippocampal atrophy, implying a potential pathway linking personality to neurodegeneration and behavior. Moreover, Waschkies et al. [61] demonstrated that combining personality assessments with CSF biomarkers significantly improved the classification of clinical AD stages, suggesting the clinical value of integrating personality assessment into different predictive models.

#### 3.3.2. Depression

Depression was the most frequently studied NPS, with 12 studies examining its associations with biomarkers and cognitive deficits. Depression was associated with AD pathology, with longitudinal studies identifying higher p-tau and low Aβ42 levels as possible factors associated with depressive symptoms [26,36,56]. Some studies further highlighted the role of elevated cortisol and altered HPA axis activity in sustaining depressive symptoms over time [47]. Findings from longitudinal studies, using MRI, revealed prefrontal and hippocampal atrophy associated with depression onset, suggesting that there are overlapping circuits with memory and emotional regulation. Cross-sectional findings supported these associations, particularly linking depression with tau burden and global atrophy in medial temporal and limbic regions [42]. However, these studies often failed to distinguish whether depression preceded, followed, or co-occurred with structural changes, limiting causal inference.

In contrast to apathy, depression was linked not only to executive dysfunction but also to memory and language impairments. Cross-sectional data confirmed these cognitive associations [33], but longitudinal findings provided more precise evidence that baseline depression predicted faster decline in cognition and increased risk of progression to AD [26,36].

#### 3.3.3. Apathy

Ten studies were focused on apathy. Longitudinal studies showed strong associations between tau pathology and executive dysfunction. Elevated p-tau and p-tau/Aβ42 ratios were significantly predictive of worsening apathy over time in multiple longitudinal investigations [26,41,59], particularly in association with degeneration in prefrontal and anterior cingulate regions. Structural imaging from these studies showed that white matter damage, especially in the cingulum and uncinate fasciculus, preceded apathy progression, supporting a model of damage in fronto-limbic circuits. Cross-sectional studies reported similar associations between apathy and tau burden [40,53], but their findings were often limited to concurrent relationships and lacked the temporal specificity that longitudinal studies provided.

Cognitively, apathy was associated with deficits in executive functions, especially in tasks involving initiation and goal setting. Cross-sectional studies often linked apathy to poor performance on executive tasks such as the Stroop and TMT-B [54]. Longitudinal studies confirmed that executive deficits may precede apathy progression [26,27,41].

#### 3.3.4. Anxiety

Anxiety was also found to be associated with biomarkers and cognitive deficits. Longitudinal studies showed that anxiety in cognitively unimpaired individuals was associated with higher plasma NfL and predicted decline in processing speed and attentional control [27,41]. These findings suggest that anxiety may signal early neural injury, even before overt tau or amyloid changes are apparent. Cross-sectional studies identified similar associations [42] and added the role of altered connectivity in salience network [67]. Furthermore, both study types identified neuroinflammatory markers (e.g., IL-6, CRP) as correlates [33]. Cognitively, anxiety was associated with deficits in attention and verbal fluency in both designs, though longitudinal data clarified that such cognitive decline may follow rather than precede anxiety [27].

#### 3.3.5. Agitation and Other Frontal Symptoms

In nine studies, frontal symptoms, including agitation, disinhibition, and irritability, were closely linked with neurodegeneration and inflammatory biomarkers. Longitudinal studies demonstrated that high levels of plasma p-tau181 and neuroinflammatory markers, such as TNF-α, were associated with the development of agitation and disinhibition [25,58] and with accelerated frontal cortical thinning and anterior cingulate degeneration [33,41,53]. Cross-sectional studies associated frontal symptoms with atrophy in the dorsolateral prefrontal cortex and white matter lesions in frontal tracts [59,64]. However, these relationships were correlational, with limited ability to account for disease progression or symptom fluctuation. Both study types supported a strong link between frontal symptoms and executive dysfunction, though only longitudinal analyses indicated that frontal network disruption predicted future behavioral dysregulation [26,41].

#### 3.3.6. Νight-Time Behavior

Analyzing five studies, night-time behavioral disturbances were shown to correlate with tau pathology, salience network dysfunction, and frontal atrophy. Longitudinal studies found that individuals with elevated p-tau/Aβ42 ratios and prefrontal cortical thinning experienced greater worsening of sleep symptoms over time [35,56,67]. APOE ε4 status was found to moderate this relationship, particularly among individuals with existing tau pathology [67]. Cross-sectional studies reported similar biomarkers but failed to prove the directional relationship. For instance, studies linking amyloid burden with fragmented sleep patterns [55] could not confirm whether sleep disturbance was a cause or consequence. In both designs, sleep disturbances were associated with hyperconnectivity in salience and default network.

#### 3.3.7. Psychotic Symptoms

In six studies, psychotic symptoms, including delusions and hallucinations, were associated with more severe AD stages. Longitudinal studies found that elevated p-tau181 and a-synuclein/Aβ42 ratio were associated with psychosis onset [52,72]. Structural MRI showed that psychosis developed in individuals with pronounced prefrontal atrophy and impaired connectivity with limbic circuits [33,41]. Cross-sectional studies confirmed these associations but were less consistent. For example, delusions were sometimes linked with hippocampal atrophy [60], but without clear differentiation between early and late disease stages. Longitudinal data provided stronger evidence that psychosis follows the accumulation of multiple pathologies and genetic susceptibility (e.g., APOE ε4) and is associated with rapid cognitive decline.

#### 3.3.8. Appetite Disorders

Only two studies in this review examined appetite changes or disorders in their outcomes. Krell-Roesch et al. (2022) found that higher levels of plasma-derived p-tau181 and p-tau217 were associated with increased symptoms of disinhibition, agitation, and appetite changes, as measured by the NPI [58]. Furthermore, Jiang et al. (2024) found that NPS can be predictors of decline in nutritional status as higher scores in NPI were longitudinally associated with lower scores in the Mini Nutritional Assessment [47]. This study also supported the idea that NPS may negatively impact eating patterns and overall nutrition, even when they are not limited to appetite-related symptoms. So, NPS such as apathy, agitation, or psychomotor dysfunction may disrupt meal routines, reduce food intake, or lead to caregiver-related feeding challenges.

## 4. Discussion

The present systematic review provides a comprehensive analysis of recent studies (2018–2024) examining the relationship between NPS in AD and three key factors: biomarkers, cognitive functions, and personality traits. Compared to previous systematic reviews, which primarily explored the role of amyloid and tau biomarkers, this study expands these findings by adding personality traits and cognitive dysfunction as possible contributors to NPS. Earlier reviews established correlations between Aβ pathology and symptoms such as apathy and depression, but they lacked extensive longitudinal evidence to assess the predictive validity of these biomarkers. Furthermore, previous research has attributed NPS to neurotransmitter imbalances, particularly in acetylcholine, serotonin, and dopamine pathways [9]. The review by Showraki et al. (2019) focused exclusively on CSF biomarkers and their association with NPS, particularly in patients with AD and MCI [77]. While agitation and aggression were the most consistently related symptoms to CSF biomarkers, it also reported conflicting results for the other NPS. The current review also includes findings on inflammatory biomarkers such as NfL and GFAP, which were not extensively covered in prior reviews but appear to be associated with NPS, particularly in early AD stages [73]. Moreover, the inclusion of both longitudinal and cross-sectional studies helped for a comparative analysis of the temporal dynamics and predictive strength of various biomarkers and cognitive domains.

Tau pathology, and especially high levels p-tau and p-tau/Aβ42 ratios, emerged as the most consistent biological factor of NPS. These biomarkers were repeatedly associated with the presence and worsening of apathy, depression, agitation, and psychosis, especially in longitudinal studies that captured their progression over time [26,30,41,56,59]. Imaging data found that tau deposition was mainly pointed in fronto-limbic circuits, confirming its role in the neural basis of NPS. In contrast, Aβ42, even though it was commonly examined, demonstrated weaker associations, especially when analyzed independently of tau. Aβ42 appears to play a more prominent role in the early stages of the disease, acting as a background condition that facilitates the emergence of NPS, rather than being a direct cause of their development [40,50,56].

Neuroinflammatory biomarkers in both CSF and plasma were also detected. Specifically, interleukin-6, C-reactive protein (CRP), NfL, and GFAP were linked with the severity of NPS [33,52,62]. Longitudinal evidence further confirmed that these inflammatory markers were not only present in symptomatic individuals but also predicted symptom onset and progression, particularly for agitation, anxiety, and frontal symptoms. Moreover, disruption in the default mode and salience networks was associated with symptoms like sleep disturbances and anxiety, especially in Aβ-positive individuals [35,55,78].

Cognitive impairment consistently related to NPS, with executive dysfunction being the most prominent cognitive dysfunction. Deficits in cognitive flexibility, attention, and inhibitory control—as assessed by tools such as the Trail Making Test-B, Stroop Test, and Symbol Digit Modalities Test—were associated with apathy, agitation, and sleep disturbances [26,41,53,54]. Importantly, longitudinal studies revealed that executive dysfunction often preceded the onset or worsening of NPS, emphasizing its predictive value. Episodic memory deficits were significantly associated with affective and psychotic symptoms [27,33,68].

While the majority of studies support strong biomarker–NPS relationships, inconsistencies have emerged, and multiple factors could explain these contradictions. One potential explanation lies in methodological differences across studies. The measurement of biomarkers varies significantly depending on the sample type (e.g., CSF vs. plasma vs. PET imaging) and the techniques used for analysis. Some studies use CSF biomarkers, which provide a more direct reflection of pathological changes in the brain, but others use plasma biomarkers, which may be affected by peripheral factors such as systemic inflammation or blood–brain barrier integrity. Additionally, variations in imaging modalities can contribute to discrepancies. For example, some studies using PET imaging have found no correlation between amyloid burden and NPS [50], whereas studies using CSF biomarkers have reported strong associations [26].

Another source of inconsistency is related to differences in patient populations. Studies included in this review ranged from individuals with MCI to mild or moderate AD, and it is likely that the role of biomarkers in NPS differs depending on the disease stage. For example, amyloid accumulation may play a more significant role in the early stages of AD, whereas tau pathology and neurodegeneration might be more strongly linked to NPS in later stages. Environmental and psychosocial factors may also account for some of the contradictory findings. NPS do not emerge solely due to underlying neuropathology but can also be influenced by lifestyle factors, social interactions, caregiver burden, and stress levels. Some studies did not control these external influences, which could contribute to variability in results.

Regarding personality traits, the analysis of the five included studies indicated that high neuroticism emerged as a risk factor for depression, anxiety, and irritability, whereas conscientiousness and extraversion demonstrated a protective effect by reducing NPS [30,43,61]. Moreover, these personality traits may interact with underlying neurobiological mechanisms. For instance, neuroticism has been associated with dysregulation of the hypothalamic–pituitary–adrenal (HPA) axis and increased cortisol levels, which have been implicated in both tau phosphorylation and progression of NPS [47]. On the other hand, extraversion and openness may reflect cognitive reserve buffering against the impact of neuropathological burden on behavior [30,61]. These findings support biopsychosocial models of dementia that integrate personality traits into the assessment and holistic treatments.

Symptom-level analysis revealed trends different associations with biomarkers and cognitive functions. Depression was strongly associated with tau pathology, prefrontal atrophy, and elevated cortisol, suggesting a synergistic effect between neurodegeneration and HPA axis dysfunction [26,36,47]. Apathy had a distinct profile, with elevated tau, fronto-limbic atrophy, and disconnection in white matter tracts such as the cingulum and uncinate fasciculus [30,41,59]. Anxiety was characterized by elevated NfL, salience network dysfunction, and neuroinflammation, appearing as both a prodromal and concurrent symptom in various stages of AD [27,41,67]. Agitation and disinhibition were linked to TNF-α, p-tau, and atrophy in the dorsolateral prefrontal cortex, reflecting advanced neurodegeneration [25,33,41]. Sleep disturbances were associated with default mode network dysregulation and tau pathology, particularly among APOE ε4 carriers [35,56]. Psychotic symptoms, including hallucinations and delusions, usually appear later in the course of the disease and were associated with α-synuclein, p-tau, and atrophy in the prefrontal cortex and limbic structures [52,60]. Appetite changes, although under-studied, were related to p-tau217 and orbitofrontal disinhibition [47,58].

A key strength of this review is the direct comparison between longitudinal and cross-sectional studies. Longitudinal designs provided stronger evidence for temporal relationships and predictive value, allowing for more definitive conclusions regarding causality and disease trajectory. Cross-sectional studies, even though they were useful for identifying patterns, were more limited by potential confounders and the inability to establish directionality.

Despite its comprehensive approach, this review has, also, some limitations. One major limitation is its biologically deterministic framework, which assumes that NPS are primarily driven by biological mechanisms such as amyloid and tau pathology, neuroinflammation, and neurotransmitter dysfunction. While biomarkers provide valuable insights into disease progression, this perspective may oversimplify the role of environmental and psychological factors in the manifestation of NPS. They can be influenced by variables such as caregiver interactions, social isolation, stress levels, and pre-existing psychiatric conditions, which were not considered in this review. Furthermore, while personality traits were included as an influencing factor, the complex interplay between psychosocial experiences and biological vulnerability remains underexplored. The timespan of this review (2018–2024) represents another limitation. While focusing on recent literature ensures the inclusion of the latest advancements in biomarker research, neuroimaging techniques, and theoretical models, it may exclude foundational studies that shaped current understanding of NPS. Another limitation relates to the heterogeneity of study methodologies, particularly in the assessment of both biomarkers and NPS. Different studies utilize CSF, PET, or plasma biomarkers, leading to inconsistent findings regarding the relationship between amyloid and tau burden with NPS. Although biomarkers are increasingly used to characterize and predict NPS, their interpretability varies based on the method of measurement (e.g., CSF, PET, plasma), disease stage, and individual-level factors such as cognitive reserve or genetic profile. While this review primarily focused on biological and cognitive correlates of NPS, future studies should adopt multimodal, prospective designs that integrate biomarkers, cognitive batteries, and psychosocial variables to generate individualized predictive models.

## 5. Conclusions

The present study highlights the complex interplay between biomarkers, cognitive functions, and personality traits in the manifestation and progression of NPS in AD. While tau pathology, neuroinflammation, and executive dysfunction emerged as prominent contributors, the expression of NPS is likely influenced by a broader set of dynamic and interrelated mechanisms. The findings support the need for longitudinal studies that integrate biological and psychosocial perspectives to better understand and manage behavioral disturbances across the continuum of dementia.

## Figures and Tables

**Table 1:** Summary of the included studies examining biomarkers, personality traits, and cognitive abilities related to NPS. The studies are presented in two parts based on their design, longitudinal or cross-sectional.

First Author, Year	Participants	Research Design	Objective of Interest	Measures	Results
Liguori, 2018 [24]	*N* = 256 (251 with AD and 55 with late life depression)	Longitudinal: 2-year follow up	To evaluate the usefulness of CSF biomarkers and FDG-PET in early differentiating late life depression and AD.	-MMSE -Patient Health Questionnaire-9 Biomarkers -18-FDG-PET -CSF Aβ42, tau, p-tau	-AD patients had lower levels of Aβ42 and reduction of 18-FDG-PET uptake in temporo-parietal regions, and the cognitive deficits were correlated with depression.
Ruthirakuhan, 2019 [25]	*N* = 38 with diagnosis of Major Neurocognitive Disorder due to AD	14-week double-blind crossover trial	To examine if serum markers of oxidative stress and neuroinflammation were associated with cannabinoid nabilone in agitated patients with AD.	-MMSE -Cohen–Mansfield Agitation Inventory Biomarkers -Lipid hydroperoxides -4-hydroxinonenal -IL-1β, IL-6, IL-8, IL-10 -Tumor necrosis factor-a (TNF-a) -Interferon	-The markers 4-hydroxynonenal and TNF-a were associated with the severity of agitation.
Banning, 2020 [26]	*N* = 24.493	Longitudinal: 5 years follow up	To examine the five-year progression of depression and apathy and their relationship to AD biomarkers.	-NPI -MMSE Biomarkers CSF Aβ42, tau, p-tau	-Lower Aβ42 and higher tau were associated with increased probability of depression and apathy over time.
Burhanullah, 2020 [27]	*N* = 470 (clinically normal older adults)	Longitudinal: follow up for approximately 5 years	To examine the relationship between NPS and subsequent cognitive decline in population-based sample.	-NPI -MMSE -Word list memory -Wechsler Logical Memory Subtest -Constructional praxis -Benton Visual Retention Test -Animal fluency -Symbol Digit Modalities Test	-Baseline NPI score predicted faster decline in word list memory, praxis recall, and animal fluency. -Baseline NPI-anxiety was associated with decline in Symbol Digit Modalities.
Huang, 2020 [28]	*N* = 793 with AD	Longitudinal: 1-year follow up	To examine how candidate gene variants relate to NPS domains.	-NPI -CDR Biomarkers -Genes	-APOE ε4 carriers displayed increased psychotic symptoms. -CD33 and EPHA1 were associated with mood symptoms. -SORL1 was associated with frontal symptoms.
Almdahl, 2021 [29]	*N* = 241 healthy older adults	Longitudinal: follow up after 4 years	To investigate potential predictors of late life depression using cognitive scores and neurodegenerative and vascular biomarkers in healthy older adults.	-NPI -MMSE -Wechsler Memory Scale, Revised Biomarkers -ΜRI -PET -CSF Aβ levels	Amyloid pathology and white matter hyperintensity can predict future development of late life depression in cognitively unimpaired individuals.
Binette, 2021 [30]	*N* = 115 older adults with family history *N* = 117 mutation carriers	Longitudinal: assessment every year	To examine the combinations of personality traits, NPS, and cognitive lifestyle related to Aβ and tau depositions.	-International Personality Item Pool NEO -NPI Biomarkers -Amyloid PET -Tau PET	-Lower neuroticism, higher openness and extraversion, lower NPS burden, and higher education were associated with less Aβ and tau depositions.
Babulal, 2022 [31]	*N* = 248 cognitively normal older adults	Longitudinal: Follow up after 3 years	To assess if baseline CSF biomarkers can predict changes in non-depressed mood states.	-The Profile of Mood States, Short Form -MMSE Biomarkers -CSF Aβ40, Aβ42, tau, and p-tau181	Participants with higher levels of CSF biomarkers developed more anxiety, anger, and fatigue over time.
Chan, 2022 [32]	*N* = 193 cognitively unimpaired older adults	Longitudinal: follow up after 3 years	To determine if amyloid burden and white matter hyperintensities modulate the association between NPS and the rate of cognitive decline.	-NPI -Geriatric Depression Scale Biomarkers -Amyloid PET	-The effect of NPS on executive dysfunction may occur through mechanisms outside of amyloid burden and white matter hyperintensity.
Clark, 2022 [33]	*N* = 87 cognitively normal or with MCI	Longitudinal: Follow up after 18–36 months	To identify systematic and central nervous system inflammatory alterations associated with NPS and their relationship with AD pathology and disease progression.	-NPI -MMSE -CDR -Buschke Double Memory Test -Stroop -Trail Making Test A and B -Activities of Daily Living Biomarkers 38 neuroinflammation and vascular injury markers in serum and CSF	-NPS were associated with eotaxin-3, IL-6, and C-reactive protein (CRP) in serum and with soluble intracellular cell adhesion molecule-1 (sICAM-1), IL-8, interferon-γ-induced protein, and CRP in CSF.
Johansson, 2022 [34]	*N* = 356 cognitively unimpaired older adults	Longitudinal: assessment biennially for up to 8 years	To examine how AD pathology biomarkers, white matter lesions, and cognitive deficits contribute to the development of apathy, anxiety, and depression.	-Apathy Evaluation Scale -Hospital Anxiety and Depression Scale -MMSE -Color/form task in a quick test Biomarkers -CSF Aβ42, Aβ40, Nfl, p-tau 217 -MRI	-Aβ pathology was associated with increasing levels of apathy and anxiety longitudinally. -More rapid decline of cognition was related to increasing levels of apathy.
Kim, 2022 [35]	*N* = 351 (with normal cognition or MCI)	Longitudinal: followed for approximately 5 years	To investigate how sleep disturbance, in combination with Aβ, tau, and APOE ε4, contributes to brain atrophy and cognitive decline.	-NPI -Informant-reported sleep disturbance (IRSD) -MMSE -Wechsler Logical Memory Subscale -Rey Auditory Verbal Learning Test -AD Assessment Schedule-Cognition -Verbal fluency -Trail Making Test A and B -Digit Span WAIS Digit Symbol -5 Clock Drawing Test -Boston Naming Test Biomarkers -CSF Aβ42 and p-tau -APOE -MRI	-Significant interaction between IRSD and AD biomarkers in multiple brain regions. -Aβ and p-tau/Aβ predicted faster decline in IRSD. -Significant interaction between IRSD and APOE for brain atrophy rate but not for cognition.
Babulal, 2023 [36]	*N* = 286 cognitively normal older adults	Longitudinal: follow up after 7 years	To investigate the effects of NPS and AD biomarkers on the progression to incident cognitive impairment among cognitively normal older adults.	-NPI -Geriatric Depression Scale -CDR Biomarkers -CSF Aβ42, Aβ40, tau, and p-tau -Amyloid PET	-Changes in NPS increase the risk of progression to cognitive impairment independently from biomarkers.
Li, 2023 [37]	*N* = 337 (167 healthy controls, 34 with SCD, 118 with MCI, 18 with AD)	Longitudinal: 1 year follow-up	To examine multimodal brain patterns associated with NPS in AD continuum.	-NPI -MMSE -MoCA -ADAS-Cog -Rey Auditory Verbal Learning Test -Categorical fluency Biomarkers -Amyloid and Tau PET -MRI and fMRI	-NPS were associated with a distinct multimodal brain network involving amyloid and tau deposition, gray matter atrophy, and functional connectivity alterations.
Marquié, 2023 [38]	*N* = 500 individuals with MCI	Longitudinal: 1 year follow up	To explore the predictive value of the combination of the AT(N) profile and NPS using survival analysis to determine the conversion ratio to dementia.	-NPI -MMSE Biomarkers -CSF Aβ, tau and p-tau protein -APOE	Pathological ATN groups and the presence of depression and apathy were associated with a higher risk of conversion to dementia.
Pink, 2023 [39]	*N* = 1581 cognitively unimpaired	Longitudinal: follow up after 15 months	To examine interactions between NPS, FDG-PET, and PiB PET.	-NPI -Auditory Verbal Learning Test -Wechsler Memory Scale -Boston Naming Test -Category fluency -Wechsler Adult Intelligence Scale -Trail Making Test A and B Biomarkers -FDG-PET -PiB PET -APOE	The combined effect of NPS and high brain amyloid leads to a faster decline in overall and specific cognitive functions.
Burling, 2024 [40]	*N* = 156 cognitively unimpaired	Longitudinal: follow up after 6–9 years	To examine the relationship between apathy and AD biomarkers in older adults.	-Apathy Evaluation Scale (Self) -Apathy Evaluation Scale (Informant) Biomarkers -PiB PET	Apathy Evaluation Scale (Informant) was significantly associated with Aβ and temporal lobe tau.
Guan, 2024 [41]	*N* = 1273 (852 with no NPS, 272 with non-MBI NPS, 147 with MBI)	Longitudinal: 1-year follow up	To investigate the association of NPS with AD structural imaging biomarkers and incident cognitive decline.	-NPI Biomarkers -MRI -APOE	-NPS were linked with hippocampal atrophy. -NPS in later life were associated with AD patterns. -MBI predicted faster progression to dementia.
Ronat, 2024 [42]	*N* = 101 healthy older adults	Longitudinal: 2-year follow up	To assess the relationship between longitudinal cognitive changes, depression, anxiety, and AD biomarkers.	-Beck Anxiety Inventory -Beck Depression Inventory -Free and Cued Selective Reminding -Trail Making Test B -3-back task -Stroop Biomarkers -Amyloid PET	-Association between anxiety and prefrontal amyloid burden classified episodic memory decline. -Depression and prefrontal and hippocampal tau burden was associated with decline in memory.
Ronat, 2024 [43]	*N* = 1286	Longitudinal: 25-year follow-up	To examine personality factors as predictors of neuropsychiatric, cognitive, and brain trajectories of participants from a population-based aging study.	-Temperament and Character Inventory -Patient Health Questionnaire-9 -Center for Epidemiologic Studies -Perceived Stress Questionnaire -Karolinska Sleep Questionnaire -Cognitive battery including 5 episodic memory scores, the WAIS-R block design test, and verbal fluency Biomarkers -MRI -APOE	-Closeness to experience and tendence to liabilities were associated with higher levels of depression, stress, sleep disturbance, and cognitive decline. -Closeness to experience was associated with faster right hippocampal volume reduction.
Rabl, 2022 [44]	*N* = 85 (with MCI or mild demntia)	Cross-sectional and longitudinal: follow up after 18–36 months	To identify blood-based biomarkers associated with NPS using untargeted plasma proteomics.	-NPI -MMSE -CDR Biomarkers -CSF Aβ42, tau, p-tau -284 plasma proteins -APOE	-The identified 15 proteins predicted both persisting NPS and cognitive decline.
Ismail, 2023 [45]	Ν = 510 MCI participants	Cross-sectional and longitudinal	To determine if adding MBI to biomarkers would improve the performance of biomarkers model.	-NPI -MMSE Biomarkers CSF Aβ42, tau, p-tau	-MBI was associated with lower Aβ42 and higher p-tau, tau, p-tau/Aβ42. -NPS were associated with lower Aβ42/Aβ40.
Ghahremani, 2023 [46]	*N* = 571 with ΜΒΙ	Cross-sectional and longitudinal (follow up after 1 year)	To investigate the associations of MBI with ptau-181, neuropsychological test performance, and incident AD.	-NPI -MMSE -Rey Auditory Verbal Learning Test -Trail Making Test B Biomarkers -Plasma p-tau181	-MBI was associated with higher plasma p-tau181 levels in addition to a decline in memory and executive functions. -Greater dementia incidence in MBI.
Jiang, 2024 [47]	Ν = 432 on Ad continuum	Cross-sectional and longitudinal: follow up after 10 months	To investigate the association between NPS and nutritional status and explore their brain regions on AD continuum.	-NPI -Mini Nutritional Assessment -MMSE -Montreal Cognitive Assessment -Activities of Daily Living -Pittsburgh Sleep Quality Index Biomarkers -APOE -Arterial spin labeling	-Increased cerebral blood flow in the left putamen was associated with malnutrition, NPS, affective symptoms, and hyperactivity. -Longitudinally, higher NPI score was associated with lower scores in Mini Nutritional Assessment.
Wang, 2019 [48]	*N* = 98 (70 with amnestic MCI, 28 with AD	Cross-sectional	To explore the neural circuits of NPS in AD.	-NPI -MMSE -Wechsler Logical Memory Subtest -CDR Biomarkers -CSF Aβ, p-tau -Resting state fMRI	A fronto-limbic circuit connects various NPS to AD pathology.
Banning, 2020 [49]	(*N* = 650) MCI (*N* = 887) AD (*N* = 626)	Cross-sectional	To investigate the relationship between AD biomarkers and NPS.	-MMSE -NPI Biomarkers -CSF Aβ42, tau protein, p-tau -MRI	-Lower levels of Aβ42 and higher levels of tau and p-tau protein were associated with anxiety. -Lower level of Aβ42 and smaller hippocampal volume were associated with apathy. -Mediation of cognitive impairment.
Sannermann, 2020 [50]	*N* = 687 (242 with SCD, 115 with MCI, 77 with AD, 209 healthy controls)	Cross-sectional	To investigate the frequency of NPS in AD subgroups and to test the association of NPS with AD biomarkers.	-NPI -Geriatric Depression Scale -Geriatric Anxiety Inventory Biomarkers -CSF Aβ42, Aβ42/Aβ40, tau, p-tau	-SCD group had less NPS compared to MCI and AD group. -In cognitively unimpaired, low Aß42 was associated with higher rates of reporting two or more NPS.
Cotta Ramusino, 2021 [51]	*N* = 100 (with MCI or dementia)	Cross-sectional	To investigate the potential correlations between NPS and CSF tau protein and brain atrophy.	-NPI -MMSE -Verbal and Digit Span -Corsi Test -15-Item Memory Test -Story Recall Test -Rey Complex Figure -Raven’s Colored Matrices -Frontal Assessment Battery -Trail Making Test A and B -Stroop Test -Verbal fluency Biomarkers -MRI -CSF Aβ42 tau, p-tau	-Negative correlation between NPI score and tau levels. -Positive correlation of cortical frontal atrophy with delusions, apathy, hallucinations, agitation, and night-time disturbances.
De Oliveira, 2021 [52]	*N* = 81(27 with Dementia with Lewy Bodies (DLB), 27 with AD and 27 controls)	Cross-sectional	To investigate associations of CSF biomarkers with neuropsychiatric features in DLB compared with late-onset AD.	-MMSE -NPI Biomarkers -Apolipoprotein E (APOE) -CSF Aβ42, Aβ40, Aβ38, tau, p-tau-181 -a-synuclein -ubiquitin -NfL	-In AD, associations of agitation with tau, tau/p-tau181, tau/Aβ42, and tau/α-synuclein. -Associations of delusions with p-tau181/Aβ42 and a-synuclein/Aβ42. -Associations of night-time behavior with tau, tau/p-tau181, tau/Aβ 42, and tau/a-synuclein.
Jacobs, 2021 [53]	*N* = 111 (60 with subjective cognitive decline, 36 with MCI, and 19 with AD)	Cross-sectional	To investigate relationships between central norepinephrine metabolism, tau and beta-amyloid, blood–brain barrier dysfunction, NPS, and memory.	-NPI -MMSE -CDR -Categorical fluency -Letter–Digit Substitution Test -Word Learning Task Biomarkers -CSF Aβ42, p-tau181 protein, albumin and 3-methoxy-4-hydroxyphenylethyleneglycol (MHPG) -Plasma (IL)1β, IL-6, IL12p70 -CSF/plasma albumin ratio	-NPS were strongly associated with MHPG and p-tau. -Memory impairment was linked to MHPG, mediated by p-tau and inflammatory amyloidosis.
Siafarikas, 2021 [54]	*N* = 145 (41 cognitively healthy, 38 late life depression, 66 predementia AD	Cross-sectional	To examine markers for synaptic function and AD pathology in late life depression.	-MMSE -Clock Drawing Test -CERAD word list test -Trail Making Test -Control Oral Speed Association Test -Visual Object and Space perception -NPI Geriatric Depression Scale Biomarkers -CSF Aβ42, Aβ40, tau, p-tau protein -Neurogranin (Ng) - Aβ precursor protein cleaving enzyme 1 (BACE 1)	-Late life depression was associated with amyloid dysmetabolism and poorer cognitive performance. -Higher Ng and BACE1 in late life depression depending on AD status.
Dang, 2022 [55]	*N* = 121 (83 with AD, 38 cognitively unimpaired)	Cross-sectional	To explore the relationship between tau, Aβ, cognition, and NPS.	-NPI -MMSE Biomarkers -FDG-PET -Amyloid PET -Tau PET	-Tau pathology is superior to Aβ and glucose metabolism to identify cognitive impairment and NPS.
Henjum, 2022 [56]	*N* = 407 (54 with MCI, 240 with AD, 113 cognitively unimpaired)	Cross-sectional	To investigate if CSF catecholamines relate to AD clinical presentation or neuropathology.	-NPI -MMSE -Clock Drawing Test -Trail Making Test A and B Biomarkers -CSF Aβ42, tau, and p-tau181 -CSF noradrenaline, adrenaline, dopamine, neurogranin,	-CSF catecholamine concentrations are altered in AD. -CSF noradrenaline and adrenaline concentrations were higher among AD patients, but their temporal dynamics may be non-linear.
Kan, 2022 [57]	*N*= 773 (cognitively healthy, MCI, dementia)	Cross-sectional	To examine the association between NPS and the burden of neurodegeneration and cerebrovascular disease and cognition.	-Frontal Assessment Battery -Digit Span -Visual Memory Span -Boston Naming Test -Verbal fluency -Word List Recall -Story Recall -Clock Drawing Test -WAIS-R block design -Symbol Digit Modality -Digit Cancellation -Maze Test -NPI Biomarkers -Quantitive MRI	-Robust association of neurodegenerative and cerebrovascular pathologies with NPS (hyperactivity and apathy) and cognitive impairment.
Krell-Roesch, 2022 [58]	*N* = 1005 (118 cognitively impaired)	Cross-sectional	To examine the associations between plasma-derived biomarkers of AD and neuropsychiatric symptoms in community-dwelling older adults.	-NPI -Short Test of Mental Status -Auditory Verbal Learning Test -Wechsler Memory Scale -Boston Naming Test -Category fluency -Trail Making Test -Wechsler Intelligence Scale -Beck Depression Inventory -Beck Anxiety Inventory Biomarkers -Plasma Aβ42/Aβ40, p-tau181, p-tau217, tau, and NfL	-p-tau181, p-tau217 and tau were associated with appetite changes. -p-tau181 and p-tau217 were associated with agitation and disinhibition.
Manca, 2022 [59]	*N* = 183 (61 with apathy, 61 with no apathy, 61 cognitively unimpaired)	Cross-sectional	To investigate the relationship between white matter damage and apathy in AD.	-NPI -MMSE -Logical Memory Test -Clock Drawing Test -Auditory Verbal Learning Test -Category fluency -Trail Making Test -Boston Naming Test Biomarkers -CSF Aβ, p-tau and ratio -MRI	-Patients with apathy have more severe NPS. -They showed signs of extensive white matter damage, especially in associative tracts in the frontal lobes, fornix, and cingulum.
Miao, 2022 [60]	Ν = 139 (86 with normal cognition and 53 with MCI)	Cross-sectional	To examine the associations between Mild Behavioral Impairment and plasma Aβ42/Aβ40.	-NPI Biomarkers -Plasma Aβ40, Aβ42	-Lower plasma Aβ42/Aβ40 was associated with higher NPI score and greater affective dysregulation.
Waschkies, 2022 [61]	*N* = 733 (189 healthy controls, 132 with amnestic MCI, and 74 with mild AD)	Cross-sectional	To evaluate the predictive value of personality traits (Big Five), anxiety and depression scores, resting-state fMRI activity of the default mode network, APOE and CSF biomarkers.	-MMSE -Geriatric Depression Scale -Geriatric Anxiety Inventory -CERAD neuropsychological battery -Big Five Inventory Biomarkers -MRI and fMRI -APOE -CSF Aβ42/40, tau, and p-tau.	CSF biomarkers, personality, depression, anxiety, and APOE show significant predictive value for classification of AD and its stages.
Aguzzoli, 2023 [62]	*N* = 109 (70 cognitively normal and 39 with cognitive impairment)	Cross-sectional	To evaluate if glial markers are associated with NPS in individuals across the AD continuum.	-NPI -MMSE -CDR Biomarkers -MRI -Amyloid PET -Tau PET -Plasma GFAP	-The NPI score and irritability were associated with microglial activation in the frontal, temporal, and parietal cortices.
De Lucia, 2023 [63]	*N* = 538 (233 with MCI, 305 healthy controls)	Cross-sectional	To evaluate the association between NPS, cognitive function, regional tau deposition, and brain volumes in MCI subjects.	-NPI -MMSE -ADAS-Cog -Trail Making Test A and B Biomarkers -MRI -Tau PET	NPS occur early in the AD trajectory and are mainly related to deficits of executive functions and to reduction of gray matter volume in the orbitofrontal and posterior cingulate cortex.
Greig Custo, 2023 [64]	*N* = 284 (55 cognitively normal, 92 with MCI and 28 with dementia)	Cross-sectional and cross-cultural	To evaluate the relationship between depression and apathy with Aβ deposition and brain atrophy.	-NPI -Geriatric Depression Scale Biomarkers -MRI -Amyloid PET	-Reduced volume in the rostral anterior cingulate cortex significantly correlated with apathy. -Apathy corresponded with higher Aβ levels.
Jiang, 2023 [65]	*N* = 310 (with MCI and AD)	Cross-sectional	To investigate the association of *APOE* ε4 with NPS and explore nutritional status and cognition as joint mediators.	-NPI -MMSE -Mini Nutritional Assessment Biomarkers -APOE	Chain-mediating effects of MNA and MMSE scores on the association of *APOE* ε4 with hallucinations, apathy, and aberrant motor activity.
Kan, 2023 [66]	*N* = 216 memory clinic participants (healthy, MCI, AD)	Cross-sectional	To examine the comorbidity of amyloid and cerebrovascular pathology with cognitive impairment and NPS.	-Wechsler Memory Scale -Frontal Assessment Battery -Digit and Visual Span -Auditory Detection Test -Boston Naming Test -Verbal fluency -Clock Drawing Test -Digit Cancellation Task -Symbol Digit Modalities Task -Maze Task Biomarkers -Structural MRI -Amyloid PET	-Negative effect of Aβ on memory and apathy. -Negative effects of cerebrovascular disease on language and hyperactivity.
Kim, 2023 [67]	*N* = 489 (53,6% cognitively normal, 32,5 with MCI, 13,9% with AD)	Cross-sectional	To examine the association between sleep disturbance, Aβ burden, and resting state functional connectivity in older adults.	-NPI -MMSE -Wechsler Logical Memory Subtest -CDR Biomarkers -Amyloid PET -Resting State fMRI	-Sleep disturbance was associated with salience hyperconnectivity, only with the presence of Aβ burden.
Krell-Roesch, 2023 [68]	*N* = 784 (699 cognitively unimpaired and 85 with MCI)	Cross-sectional	To examine the association between CSF biomarkers and NPS in older non-demented adults.	-NPI -Beck Depression Inventory -Beck Depression Anxiety Biomarkers CSF Aβ42, tau, p-tau181	Lower CSF Aβ42 and higher tau/Aβ42 and p-tau/Aβ42 ratios were associated with depression and anxiety, as well as with NPI-assessed anxiety, apathy, and night-time behavior.
Ozaki, 2023 [69]	*N* = 122 (12 cognitively unimpaired, 46 with MCI and 64 with AD)	Cross-sectional	To analyze the relationship between p-tau protein and depression, anxiety, and apathy.	-NPI -MMSE Biomarkers -CSF p-tau protein -APOE	-Association between p-tau accumulation and decreased incidence of depression and apathy. -In APOE ε4 non-carriers: negative association between p-tau and depression.
Frank, 2024 [70]	*N* = 781 older adults (218 with dementia)	Cross-sectional	To analyze cross-sectional mediation pathways between CSF biomarkers, cognitive function, and NPS.	-NPI -MMSE MoCA -Logical Memory Recall -Boston Naming Test -Semantic fluency -Trail Making Test B Biomarkers CSF Aβ42, tau, p-tau181	Higher p-tau181/Aβ42 ratio predicted higher NPI score, which was partially mediated by the MMSE and MoCA.
Falgas, 2024 [71]	Ν = 136 (104 with AD and 32 healthy controls)	Cross-sectional	To determine the differences in the severity of NPS and locus coeruleus.	-NPI Biomarkers -CSF Aβ42, tau, p-tau, and noradrenaline -Plasma p-tau181 -MRI	-Early onset AD was associated with higher NPI score. -Locus coeruleus integrity was negatively associated with NPS. -Noradrenaline levels increased in AD.
Huang, 2024 [72]	*N* = 305 (53 normal controls, 75 with subjective cognitive decline, 74 with MCI, 103 with dementia)	Cross-sectional	To explore the prevalence of NPS and their association with plasma biomarkers throughout the Alzheimer’s continuum.	-NPI Biomarkers Plasma Aβ42, Aβ40, tau, p-tau181, NfL, APOE	-NPS can be early manifestations of preclinical AD. - Higher plasma NfL levels seem to be associated with NPS and especially psychosis.
Hsu, 2024 [73]	*Ν* = 1896 (977 cognitively unimpaired, 270 with MCI and 649 with dementia)	Cross-sectional	To investigate the association of NPS with CSF biomarkers across a spectrum of cognitive states.	-NPI Biomarkers CSF Aβ42, tau, and p-tau181 protein	-The notable disparities in NPI and CSF biomarkers among normal, MCI, and AD patients underscore their diagnostic potential.

NPS = neuropsychiatric symptoms, NPI = neuropsychiatric inventory, AD = Alzheimer’s disease, MCI = Mild Cognitive Impairment, MBI = Mild Behavioral Impairment, SCD = subjective cognitive decline, MMSE = Mini-Mental State Examination, CDR = Clinical Dementia Rating, CSF = cerebrospinal fluid, Aβ = amyloid β, tau = tau protein, p-tau = phospho-tau protein, PET = Positron Emission Tomography, PiB PET = Pittsburgh Compound B PET, FDG-PET = fluorodeoxyglucose Positron Emission Tomography, MRI = Magnetic Resonance Imaging, fMRI = Functional MRI, APOE = Apolipoprotein E, NfL = Neurofilament Light, IL = interleukin.

**Table 2:** Summary of biomarkers, cognitive functions, imaging data, and personality trait correlates associated with specific NPS.

Symptom	Key Biomarkers	Main Cognitive Domains	Imaging Findings	Personality Traits
Apathy	↑ p-tau, ↑ p-tau/Aβ42	Executive dysfunction	Atrophy: anterior cingulate, prefrontal cortex; white matter loss in cingulum, uncinate	↓ extraversion, ↓ conscientiousness
Depression	↑ p-tau, ↑ cortisol, ↓ Aβ42	Memory, language, global cognition	Atrophy: hippocampus, medial prefrontal cortex; ↑ tau in hippocampus	↑ neuroticism, ↓ conscientiousness
Anxiety	↑ NfL, ↑ IL-6, ↑ CRP	Attention, processing speed	Hyperconnectivity in salience network; amyloid in prefrontal cortex	↑ neuroticism
Agitation/Frontal Symptoms	↑ p-tau181, ↑ TNF-α, ↑ oxidative stress	Executive control	Thinning: dorsolateral prefrontal cortex, anterior cingulate; microglial activation in frontal cortex	
Sleep Disturbance	↑ p-tau/Aβ42, APOE ε4 x Aβ interaction	Arousal regulation, attention	Altered default mode network, ↑ tau in precuneus; frontal cortical thinning	
Psychosis	↑ a-syn/Aβ42, ↑ p-tau, ↑ NfL	Working memory, attention	Atrophy: prefrontal cortex, limbic structures; connectivity disruption	
Appetite Disturbance	↑ p-tau217, ↓ Aβ42, ↑ inflammatory markers	Executive disinhibition	Atrophy: hypothalamus, orbitofrontal cortex	

↑ = high or elevated levels, ↓ = low levels.

## Data Availability

The original contributions presented in this study are included in the article. Further inquiries can be directed to the corresponding author.

## Appendix A

**Table A1:** Scores of included studies on Newcastle–Ottawa Scale.

	Selection				Comparability		Outcome			
Study	Representative of the Exposed Cohort	Selection of Non-Exposed Cohort	Ascertainment of Exposure	Outcome of the Interest Not Present at the Start of the Study	Main Factor	Additional Factor	Assessment of Outcomes	Sufficient Follow-Up Time	Adequacy of Follow-Up	Total
**Liguori, 2018 [24]**	*	*	*	*	*	*	*	*	*	9/9
**Ruthirakuhan, 2019 [25]**	-	-	*	*	*	*	*	*	*	7/9
**Banning, 2020 [26]**	*	*	*	*	*	*	*	*	*	9/9
**Burhanullah, 2020 [27]**	*	*	*	*	*	*	*	*	*	9/9
**Huang, 2020 [28]**	*	*	*	*	*	*	*	*	*	9/9
**Almdahl, 2023 [29]**	*	*	*	*	*	*	*	*	*	9/9
**Binette, 2021 [30]**	*	*	*	*	*	*	*	*	-	8/9
**Babulal, 2022 [31]**	*	*	*	*	*	-	*	*	*	8/9
**Chan, 2022 [32]**	*	*	*	*	*	*	*	*	*	9/9
**Clark, 2022 [33]**	-	-	*	*	*	*	*	*	*	7/9
**Johansson, 2022 [34]**	*	*	*	*	*	*	*	*	*	9/9
**Kim, 2022 [35]**	*	*	*	-	*	*	*	*	-	7/9
**Babulal, 2023 [36]**	*	*	*	*	*	*	*	*	-	8/9
**Li, 2023 [37]**	*	*	*	*	*	*	*	*	*	9/9
**Marquié, 2023 [38]**	*	*	*	*	*	*	*	*	*	9/9
**Pink, 2023 [39]**	*	-	*	*	*	*	*	*	*	8/9
**Burling, 2024 [40]**	*	*	*	*	*	*	*	*	*	9/9
**Guan, 2024 [41]**	*	*	*	*	*	*	*	*	*	9/9
**Ronat, 2024 [42]**	*	*	*	*	*	*	*	*	*	9/9
**Ronat, 2024 [43]**	*	*	*	*	*	*	*	*	*	9/9
**Rabl, 2022 [44]**	-	-	*	*	*	*	*	*	*	7/9
**Ismail, 2023 [45]**	*	*	*	*	*	*	*	*	*	9/9
**Ghahremani, 2023 [46]**	*	*	*	*	*	*	*	*	*	9/9
**Jiang, 2024 [47]**	*	*	*	*	*	*	*	*	*	9/9
**Wang, 2019 [48]**	*	-	*	-	*	*	*	-	-	5/9
**Banning, 2020 [49]**	*	-	*	-	*	*	*	-	-	5/9
**Sannermann, 2020 [50]**	*	*	*	-	*	*	*	-	-	6/9
**Cotta Ramusino, 2021 [51]**	*	-	*	-	*	*	*	-	-	5/9
**De Oliveira, 2021 [52]**	*	*	*	-	*	*	*	-	-	6/9
**Jacobs, 2021 [53]**	*	*	*	-	*	*	*	-	-	6/9
**Siafarikas, 2021 [54]**	*	*	*	-	*	*	*	-	-	6/9
**Dang, 2022 [55]**	*	*	*	-	*	*	*	-	-	6/9
**Henjum, 2022 [56]**	*	-	*	-	*	*	*	-	-	5/9
**Kan, 2022 [57]**	*	*	*	-	*	-	*	-	-	5/9
**Krell-Roesch, 2022 [58]**	*	-	*	-	*	*	*	-	-	5/9
**Manca, 2022 [59]**	*	*	*	-	*	*	*	-	-	6/9
**Miao, 2022 [60]**	*	*	*	-	*	*	*	-	-	6/9
**Waschkies, 2022 [61]**	*	*	*	-	*	*	*	-	-	6/9
**Aguzzoli, 2023 [62]**	*	*	*	-	*	*	*	-	-	6/9
**De Lucia, 2023 [63]**	*	-	*	-	*	*	*	-	-	5/9
**Greig Custo, 2023 [64]**	*	*	*	-	*	*	*	-	-	6/9
**Jiang, 2023 [65]**	*	-	*	-	*	*	*	-	-	5/9
**Kan, 2023 [66]**	*	*	*	-	*	*	*	-	-	6/9
**Kim, 2023 [67]**	*	*	*	-	*	*	*	-	-	6/9
**Krell-Roesch, 2023 [68]**	*	*	*	-	*	*	*	-	-	5/9
**Ozaki, 2023 [69]**	-	*	*	-	*	*	*	-	-	5/9
**Falgas, 2024 [70]**	*	*	*	-	*	*	*	-	-	6/9
**Frank, 2024 [71]**	*	-	*	-	*	*	*	-	-	5/9
**Huang, 2024 [72]**	*	*	*	-	*	*	*	-	-	5/9
**Hsu, 2024 [73]**	*	*	*	-	*	*	*	-	-	6/9

* = The study fulfills the criterion, - = The study does not fulfill the criterion.
